# Tumor Treating Fields (TTFields) sensitize anti-PD1-resistant lung tumors to PD1 blockade and remodel myeloid and lymphoid states

**DOI:** 10.21203/rs.3.rs-10539539/v1

**Published:** 2026-08-06

**Authors:** Yun Hu, Fatemeh Maspourour, Qi Wang, Ailing Huang, Hampartsoum Barsoumian, Zahid Rafiq, Ashley Hoffman, Denton Robinson, Jing Wang, James W. Welsh

**Affiliations:** The University of Texas MD Anderson Cancer Center; The University of Texas MD Anderson Cancer Center; The University of Texas MD Anderson Cancer Center; The University of Texas MD Anderson Cancer Center; The University of Texas MD Anderson Cancer Center; The University of Texas MD Anderson Cancer Center; The University of Texas MD Anderson Cancer Center; The University of Texas MD Anderson Cancer Center; The University of Texas MD Anderson Cancer Center; The University of Texas MD Anderson Cancer Center

**Keywords:** Tumor Treating Fields, PD1 blockade resistance, lung adenocarcinoma, tumor immune microenvironment, macrophage reprogramming

## Abstract

**Background:**

Primary resistance to programmed cell death protein 1 (PD1) blockade is sustained by defective antigen presentation, immunosuppressive myeloid programs, regulatory T-cell (Treg) dominance, and dysfunctional lymphocyte states. Tumor Treating Fields (TTFields) are non-ionizing alternating electric fields with established antiproliferative activity; however, whether they can overcome resistance to immune checkpoint blockade remains unclear.

**Methods:**

The antitumor and immunomodulatory effects of TTFields were investigated in anti-PD1-resistant 344SQR murine lung adenocarcinoma cells and syngeneic tumors. Cells were exposed to TTFields for 24, 48, or 72 h and evaluated for proliferation, clonogenic survival, and transcriptional pathway changes. Tumor-bearing mice received control treatment, anti-PD1, matched heating control, TTFields, or their combinations. Tumor growth, survival, and pulmonary metastatic burden were assessed, and tumor-infiltrating immune populations were characterized by single-cell RNA sequencing and T-cell receptor clonotype analysis.

**Results:**

TTFields suppressed tumor-cell proliferation and clonogenic survival in a time-dependent manner, with maximal inhibition at 48 h and partial recovery at 72 h. This biphasic response coincided with early suppression followed by reactivation of cell-cycle, metabolic, and interferon-related transcriptional programs. Anti-PD1 monotherapy showed minimal activity in vivo, whereas TTFields combined with anti-PD1 delayed tumor growth, reduced pulmonary metastases, and prolonged survival. Single-cell analysis showed that TTFields decreased Treg abundance, increased the CD8^+^ T-cell/Treg ratio, and shifted tumor-associated macrophages toward antigen-presenting and inflammatory states characterized by increased Cd86, H2-Ab1, Nos2, and Tnf and decreased Mrc1 and Tgfb1 expression. Combination treatment enriched Gzmk-dominant CD8^+^ T-cell and natural killer T-cell states, reduced Pdcd1 and Ctla4 expression, and promoted focused expansion of dominant T-cell clonotypes.

**Conclusions:**

TTFields sensitized anti-PD1-refractory lung tumors to PD1 blockade and induced coordinated remodeling of tumor-cell fitness and the tumor immune microenvironment. These findings identify TTFields as a potential tumor-conditioning strategy for overcoming resistance to immune checkpoint blockade.

## Background

Immune checkpoint inhibitors (ICIs) have transformed the treatment landscape of solid tumors, yet durable clinical benefit remains limited to a subset of patients [1, 2]. Resistance to PD1-directed therapy arises from dysfunction across multiple levels of the tumor-immune ecosystem, including insufficient innate immune priming, impaired antigen presentation, persistent immunoregulatory dominance and maladaptive lymphocyte states marked by sustained inhibitory receptor expression [2–5]. In this setting, simply intensifying checkpoint inhibition is often insufficient, highlighting the need for strategies that reprograms tumor immune interactions more broadly and restore conditions permissive for effective antitumor immunity.

Radiotherapy has provided proof of principle that local tumor perturbation can enhance systemic immune responses and improve sensitivity to immunotherapy [6–8]. However, radiation-based treatment regimens also have important limitations, including lymphodepletion, induction of compensatory immunosuppressive pathways such as TGF-β signalling, and practical constraints related to dose, anatomy and cumulative toxicity [9, 10]. These liabilities underscore the need for alternative tumor-conditioning approaches that can reshape the immune composition and function without relying on ionizing damage.

Tumor Treating Fields (TTFields) are a non-ionizing biophysical modality that delivers low intensity, intermediate frequency alternating electric fields to disrupt mitotic processes in dividing tumor cells [11, 12]. While TTFields are best known for their direct antiproliferative effects, emerging evidence suggests that they may also influence tumor-cell state and thereby remodel the immune microenvironment [13, 14]. However, the extent to which TTFields can overcome resistance to PD1 blockade, and the immunological mechanisms underlying this effect, remain incompletely defined.

To answer this, we used an anti-PD1-refractory 344SQR lung adenocarcinoma model [15], to investigate whether TTFields function not only as a tumor-directed therapy but also as an immune-conditioning strategy. We show that TTFields induce dynamic tumor-cell reprogramming, with early suppression of proliferative pathways followed by partial transcriptional adaptation during prolonged exposure. In vivo, TTFields restore responsiveness to PD1 blockade and improve tumor control, reduce metastatic burden and extend survival. Single-cell and TCR analyses further reveal coordinated immune remodelling characterized by an increased CD8^+^ T cell-to-Treg ratio, enhanced macrophage antigen-presentation and pro-inflammatory polarization. Additionally, we observed reduced Tgfb1-associated immunosuppressive features, attenuation of inhibitory checkpoint architecture in lymphocytes, and focused expansion of dominant T cell clonotypes. Together, these findings position TTFields as a mechanistically distinct tumor-conditioning modality that overcomes checkpoint resistance through integrated reprogramming of tumor-intrinsic, innate and adaptive immune states.

## Methods

### Cell lines and culture conditions

The anti-PD1-resistant murine lung adenocarcinoma cell line 344SQR was previously generated in our laboratory from the parental 344SQ line through in vivo selection under PD1 blockade [15]. Cells were maintained in complete RPMI-1640 medium supplemented with 10% heat-inactivated fetal bovine serum and 100 U ml^−1^ penicillin–streptomycin. Cultures were incubated at 37°C in a humidified atmosphere containing 5% CO_2_.

### In vitro TTFields treatment

TTFields were delivered using the Inovitro^™^ system (Novocure) according to the manufacturer’s instructions. Briefly, electric field parameters were set to 150 kHz, and temperature within the ceramic dishes was maintained at 37°C throughout exposure. Sterile coverslips were placed in each TTFields ceramic dish according to the manufacturer’s protocol. For proliferation assays, 344SQR cells were seeded at 1 × 10^4^ cells per well. Cells were allowed to adhere overnight, after which 2 ml of fresh complete medium was added and plates were placed into the TTFields device. Cells were exposed continuously to TTFields for 24, 48 or 72 hours. Time-matched untreated plates maintained at 37°C served as controls. At each time point, cells were harvested by trypsinization, and total cell number was counted. Relative cell percentage was calculated by normalizing experimental conditions to corresponding untreated controls.

### RNA extraction and bulk RNA sequencing

Cells were collected after 48 or 72 h of TTFields exposure and pelleted by centrifugation at 1,500 rpm for 7 min at 4°C. Total RNA was extracted using TRIzol according to the manufacturer’s instructions. RNA concentration and quality were assessed on a NanoDrop 2000 before library preparation.

Strand-specific, Illumina-compatible total RNA libraries were generated from 100 ng DNase I-treated total RNA using the Illumina Stranded Total RNA Prep Ligation with Ribo-Zero Plus kit. Cytoplasmic and mitochondrial ribosomal RNA were depleted using Ribo-Zero Plus probe-based chemistry. The remaining RNA was fragmented and reverse transcribed with random hexamers, and dUTP was incorporated during second-strand synthesis to preserve strand information. Blunt-ended double-stranded cDNA fragments were then 3′ A-tailed, ligated to pre-index anchors, purified, and amplified by 13 cycles of PCR to introduce dual indexes.

Libraries were quantified using the Qubit dsDNA HS Assay Kit and assessed for fragment size distribution on an Agilent TapeStation. Individual libraries were pooled (14 libraries per pool), quantified by qPCR using the KAPA Library Quantification Kit, and sequenced on a single lane of an Illumina NovaSeq X 1.5B-200 flow cell in 100-bp paired-end mode.

Differential expression analysis and gene set enrichment analysis were performed relative to untreated controls to define transcriptional programs altered by TTFields exposure.

### Clonogenic survival assay

To assess long-term reproductive viability after TTFields exposure, clonogenic assays were performed. Following 24, 48, or 72 hours of treatment, cells were harvested and replated at 500 cells per well in standard 6-well tissue culture plates containing complete growth medium. Cells were then maintained under standard culture conditions for approximately 6 days to permit colony formation. Colonies were fixed with ethanol, stained with crystal violet, and scanned using an Epson Expression 10000XL scanner in Professional mode. Colonies were quantified in ImageJ, and surviving fraction was determined by normalizing colony numbers in TTFields-treated samples to the corresponding untreated controls at each time point.

### In vivo tumor model and treatment

Female 129Sv/Ev syngeneic mice (8–10 weeks of age) were subcutaneously inoculated in the right flank with 5 × 10^4^ 344SQR cells. Once tumors were established, mice were randomized (n = 6–12 per group) into six treatment arms: (1) control; (2) anti-PD1; (3) heating; (4) heating plus anti-PD1; (5) TTFields; and (6) TTFields plus anti-PD1.

TTFields (150 kHz; target temperature 38.5°C) or heating control were initiated on day 4 after tumor implantation and delivered continuously for 7 consecutive days. Anti-PD1 antibody (BioXcell, Cat.#BE0146, 200 μg per dose) was administered intraperitoneally on days 11, 15, 18, 21 and 28. Tumor dimensions were measured with calipers, and volumes were calculated as V = 0.5 × (width^2^ × length). Mice were euthanized when tumors reached 14 mm in maximal diameter. All procedures were performed in accordance with protocols approved by the Institutional Animal Care and Use Committee of MD Anderson Cancer Center.

### Single-cell RNA sequencing (scRNAseq)

Tumors were harvested on day 20 after inoculation and processed for scRNAseq as previously described [16, 17]. Briefly, tumors were minced and digested with 250 μg/mL Liberase (Cat. #05401127001, Roche) and 20 μg/mL DNase I (Cat. #4716728001, Sigma-Aldrich) at 37°C for 30 min. Digestion was quenched with fetal bovine serum, and the resulting cell suspensions were filtered. Cells from mice within the same treatment group were pooled, stained with anti-CD45-FITC, washed in RPMI 1640 containing 2% fetal bovine serum, and sorted on a BD FACSAria II cell sorter. For each sample, at least 1 ×10^5^ CD45^+^ cells with > 85% viability were used for downstream analysis.

Single-cell library preparation was performed according to the 10x Genomics Chromium Next GEM 5′ single-cell RNA-seq v2 and TCR enrichment protocols. Following cDNA amplification and library preparation, library quality and concentration were assessed using the Qubit High-Sensitivity dsDNA Assay and Agilent High-Sensitivity DNA Bioanalyzer. Library concentrations were further confirmed by qPCR using the KAPA Library Quantification Kit. Samples were normalized to 5 nM and pooled at a 5:1 ratio of gene expression library to TCR library. Sequencing was performed on an Illumina NovaSeq 6000 S2 100-cycle flow cell.

Raw unique molecular identifier count matrices were analyzed using Seurat (v4.1.0) in R. Cells with > 25% mitochondrial transcripts or lacking Ptprc expression were excluded. Data were log-normalized with a scale factor of 10,000, and the four samples were integrated using the top 2,000 highly variable genes. Principal component analysis was performed on the integrated dataset, and cell clusters were identified using Seurat clustering at a resolution of 0.5 and visualized by UMAP.

Cell identities were assigned using the ImmGenData reference dataset in SingleR together with manual curation. Because all sequenced cells were CD45^+^, nonimmune reference cell types were excluded from annotation. T cell populations were further resolved into CD4^+^ and CD8^+^ subsets. Conserved marker genes were used to confirm cluster identities, and differentially expressed genes between clusters and treatment groups were identified using Seurat marker analysis functions.

### Statistical analysis

Statistical analyses were performed as previously described [16–18]. Tumor volumes were analyzed using two-way analysis of variance (ANOVA). Overall survival was estimated by the Kaplan-Meier method and compared using two-sided log-rank tests. Lung metastatic burden was evaluated using two-tailed unpaired t-tests. scRNAseq data were analysed using the Kruskal-Wallis test. All reported values represent mean ± S.E.M., and a two-sided P < 0.05 was considered statistically significant.

## Results

### TTFields Induces Time-Dependent Growth Inhibition in 344SQR cells

**Fig. 1:**
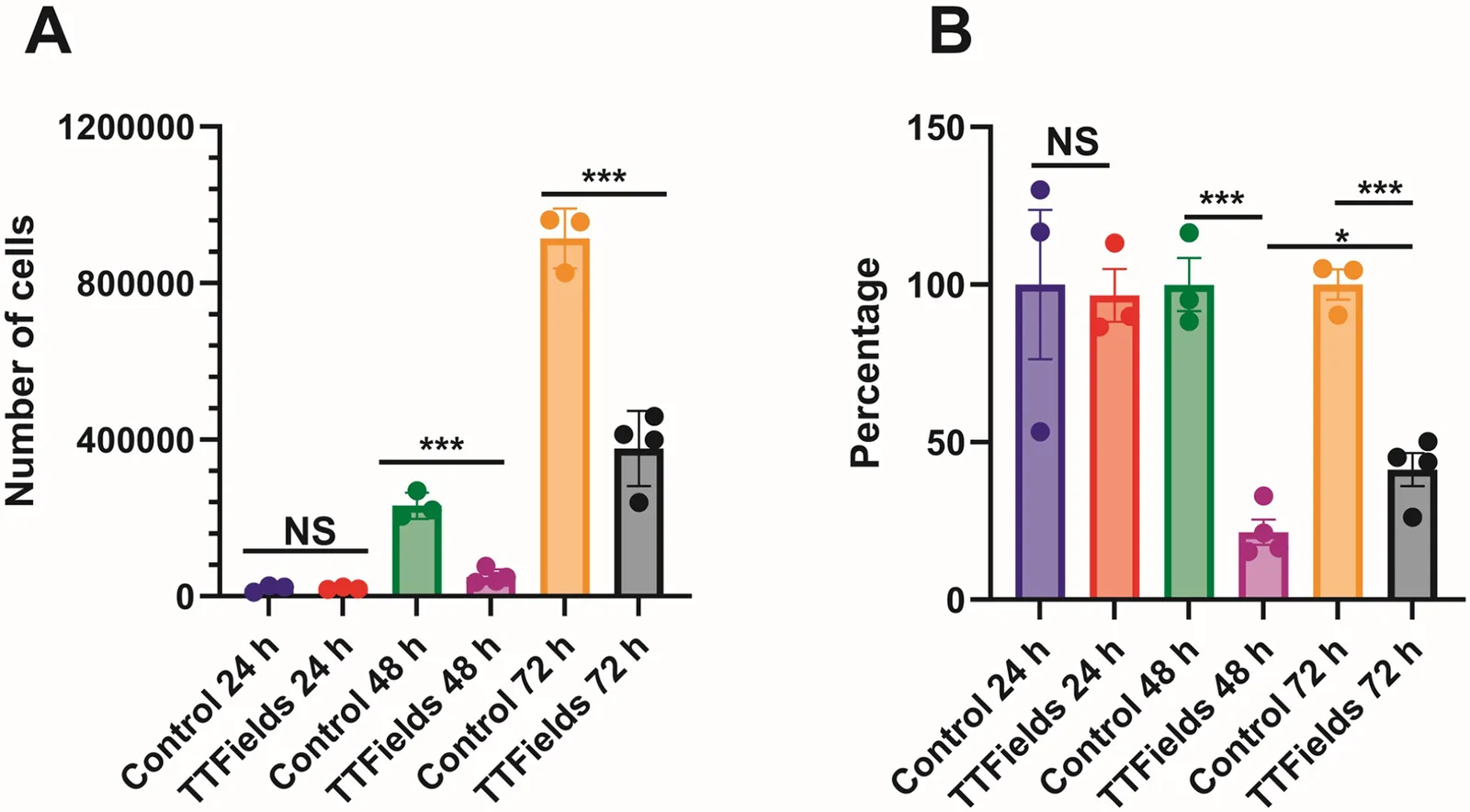
TTFields induce time-dependent growth inhibition in 344SQR cells. 344SQR murine lung adenocarcinoma cells were treated in vitro with TTFields using the inovitro^™^ system at 150 kHz, with temperature maintained at 37 °C. Cells were seeded at 1 × 10^4^ per well on sterile coverslips placed in ceramic dishes, allowed to adhere overnight, and then exposed continuously to TTFields for 24, 48 or 72 h. Time-matched untreated plates maintained under identical incubator conditions served as controls. At each time point, cells were harvested and quantified. **A** Total cell number at each time point. **B** Cell viability expressed as percentage of untreated controls. Data are presented as mean ± SEM. NS, not significant; *P < 0.05; ***P < 0.001.

To define the direct antiproliferative activity of TTFields in an anti-PD1-refractory setting, 344SQR cells were exposed in vitro for 24, 48 or 72 h alongside time-matched untreated controls. At 24 h, TTFields did not significantly alter total cell number (Fig. 1A) or normalized cell percentage (Fig. 1B), indicating minimal early growth inhibition. By 48 h, however, TTFields induced significant suppression of tumor expansion, reducing absolute cell counts from 231,000 ± 19,553 in controls to 49,550 ± 9,294 under field exposure, corresponding to a decline in normalized cell percentage from 100% ± 8.47% to 21.45% ± 4.02%. This time point represented the maximal antiproliferative effect observed.

At 72 h, TTFields-treated cultures remained significantly growth-inhibited compared with controls (377,500 ± 47,912 versus 914,333 ± 44,190), but the magnitude of suppression was attenuated relative to 48 h, with normalized cell percentage increasing to 41.29% ± 5.24%. Thus, although TTFields sustained significant growth restraint at prolonged exposure, the relative reduction in proliferative output was diminished compared to the 48 h time point. This biphasic response suggests the emergence of adaptive transcriptional or phenotypic programs, or selection of less sensitive subpopulations, enabling partial proliferative recovery during continued field exposure.

### TTFields Reduces Long-Term Clonogenic Survival of 344SQR cells

**Fig. 2:**
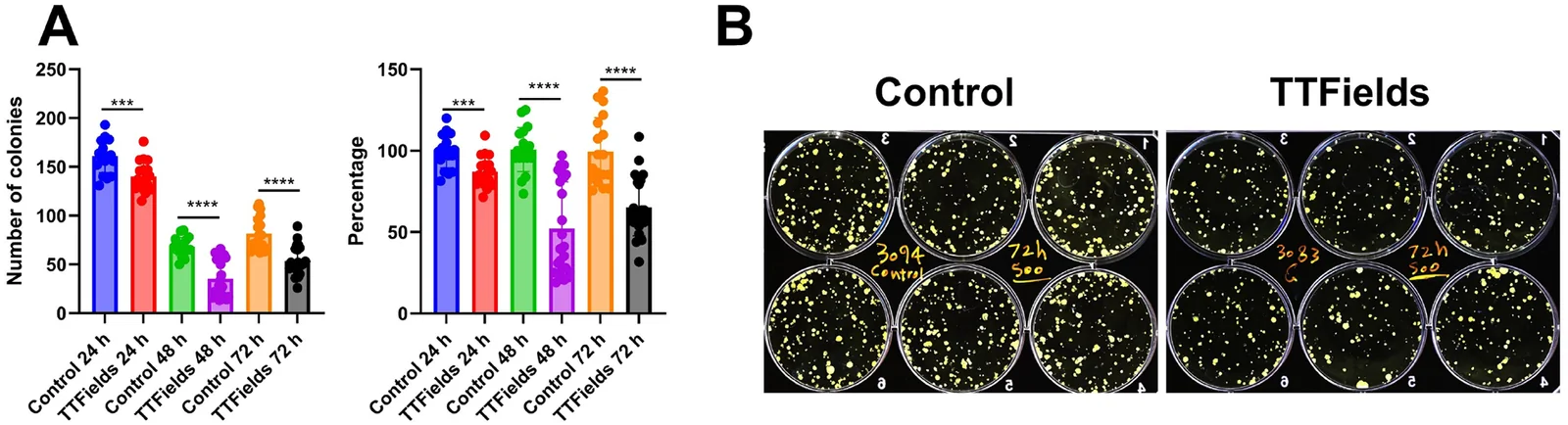
TTFields reduce long-term clonogenic survival of 344SQR cells. 344SQR cells were exposed to TTFields for 24, 48 or 72 h, after which 500 cells per well were replated in standard 6-well plates containing complete medium and cultured for 6 days to permit colony formation. Colonies were fixed with ethanol, stained with crystal violet and counted manually. Surviving fraction was calculated by normalizing colony numbers in treated groups to time-matched untreated controls. **A** Quantification of colony number and surviving fraction expressed as percentage of control. **B** Representative clonogenic images comparing TTFields-treated and control groups. Data are presented as mean ± SEM. NS, not significant; ***P < 0.001; ****p < 0.0001.

Because short-term growth effects may underestimate durable cytotoxicity, we next assessed clonogenic survival following TTFields exposure. After 24, 48 or 72 h treatment, 500 viable cells were reseeded and cultured for 6 days before colony staining; surviving fraction was normalized to time-matched controls. As shown in Fig. 2A, TTFields induced a modest but significant decrease in clonogenic output after 24 h (87.13% of control), followed by a significantly larger impairment after 48 h (52.21% of control). At 72 h, as shown in Fig. 2A and 2B, clonogenic survival remained significantly reduced (65.14% of control) but exceeded the suppression observed at 48 h, again indicating partial recovery with prolonged exposure. Together with the short-term growth measurements, these results establish a biphasic response in which TTFields maximally impair both proliferation and reproductive viability at 48 h, followed by partial rebound in cancer cell viability at 72 h.

### Dynamic Transcriptional Reprogramming Following TTFields exposure

**Fig. 3:**
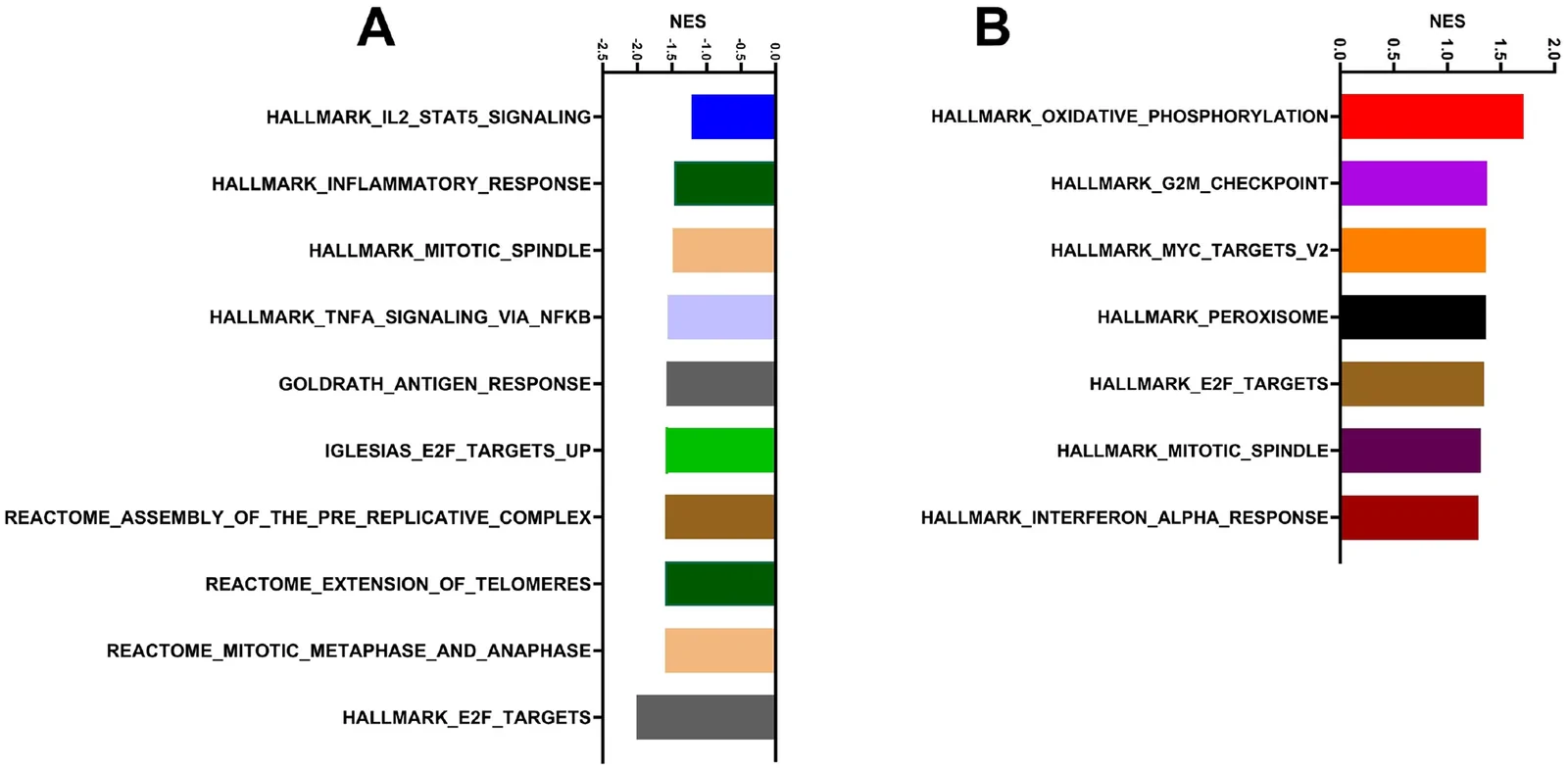
TTFields induce time-dependent transcriptional changes revealed by GSEA. 344SQR tumor cells were exposed to TTFields and subjected to RNA sequencing after 48 h or 72 h of treatment. GSEA was performed comparing TTFields-treated cells with untreated controls. **A** Enriched pathways at 48 h following TTFields exposure. **B** Enriched pathways at 72 h following TTFields exposure. Bars represent normalized enrichment scores (NES) for significantly enriched gene sets. Positive NES indicates pathways enriched in TTFields-treated cells, whereas negative NES indicates pathways enriched in untreated controls.

To connect this biphasic phenotype to underlying tumor cell state, we performed RNA-seq and gene set enrichment analysis (GSEA) to compare 48 h and 72 h TTFields exposure relative to untreated controls. At 48 h, TTFields induced broad negative enrichment of cell-cycle and mitotic pathways, including HALLMARK_MITOTIC_SPINDLE and HALLMARK_E2F_TARGETS, together with Reactome pathways linked to mitotic progression and pre-replicative complex assembly (Fig. 3A and Supplemental Fig. 1). Oncogenic transcriptional outputs were also suppressed, including HALLMARK_MYC_TARGETS_V1 and HALLMARK_KRAS_SIGNALING_UP. Notably, TTFields concurrently downregulated inflammatory and survival-associated modules: HALLMARK_INFLAMMATORY_RESPONSE, HALLMARK_TNFA_SIGNALING_VIA_NFKB, HALLMARK_IL6_JAK_STAT3_SIGNALING and HALLMARK_APOPTOSIS-consistent with a dominant anti-proliferative state accompanied by dampened stress–inflammatory signalling at this time point. In contrast, the 72 h profile showed a marked inversion of the 48 h signature (Fig. 3B). Core proliferative pathways shifted to positive enrichment, including HALLMARK_G2M_CHECKPOINT, HALLMARK_E2F_TARGETS, HALLMARK_MYC_TARGETS_V2 and HALLMARK_MITOTIC_SPINDLE, indicating reactivation of cell-cycle machinery. Metabolic pathways, HALLMARK_OXIDATIVE_PHOSPHORYLATION, HALLMARK_PEROXISOME and HALLMARK_HEME_METABOLISM were also positively enriched, suggesting restoration or enhancement of oxidative metabolism during prolonged exposure. In parallel, HALLMARK_INTERFERON_ALPHA_RESPONSE became positively enriched at 72 h, indicating re-engagement of interferon-associated signalling. Thus, TTFields trigger an early transcriptional shutdown of proliferative and associated signalling programs that is followed by transcriptional rebound, providing a molecular correlate for the reduced magnitude of growth inhibition and improved clonogenic capacity observed at 72 h.

### TTFields restore responsiveness to PD1 blockade and extend survival in vivo

**Fig. 4:**
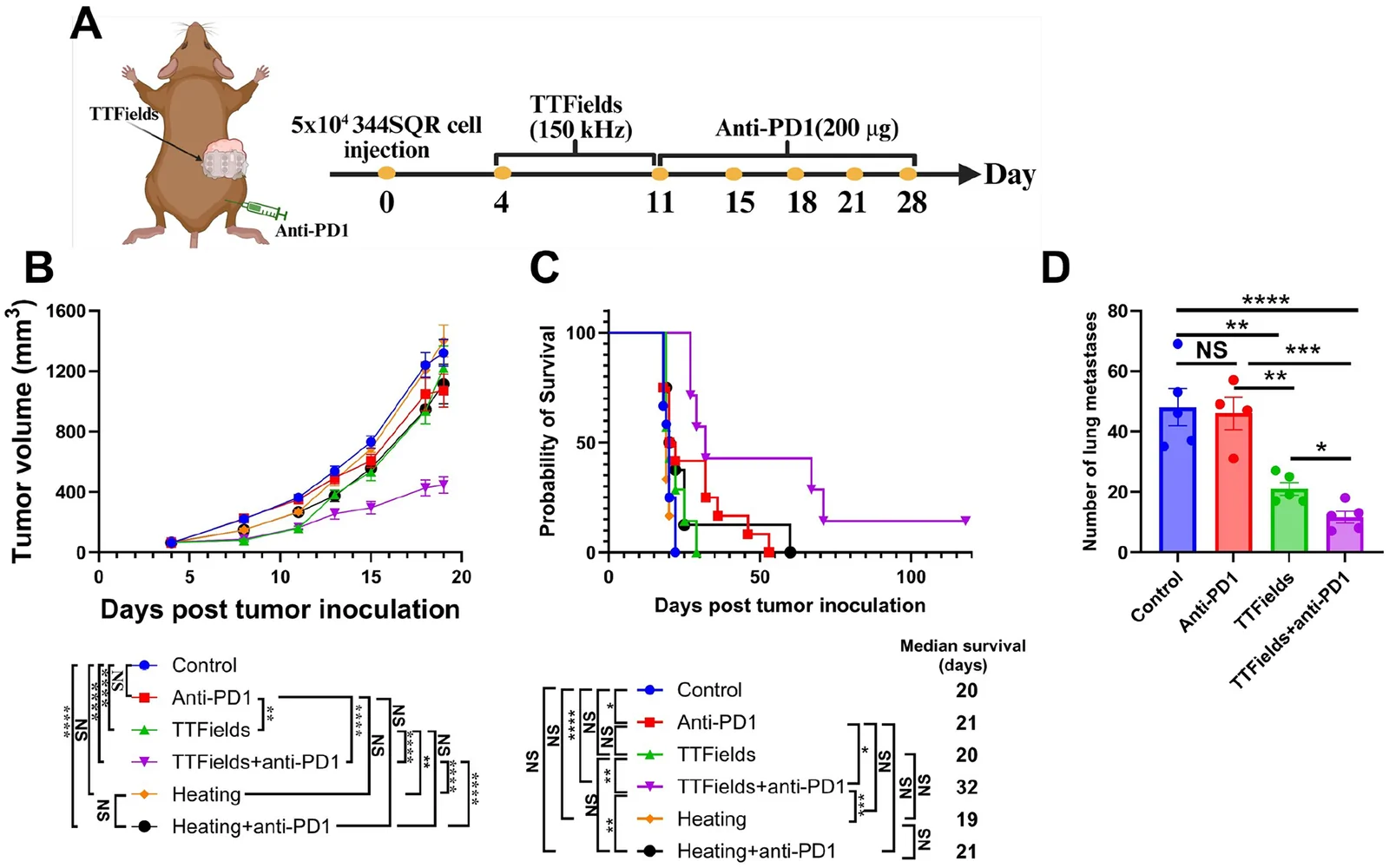
TTFields restore responsiveness to PD-1 blockade and prolong survival in vivo. Female syngeneic mice (8–10 weeks) were inoculated subcutaneously with 5 × 10^4^ 344SQR cells and randomized (n = 6–12 per group) to control, anti-PD1, heating, heating + anti-PD1, TTFields, or TTFields + anti-PD1. TTFields (150 kHz; target temperature 38.5 °C) or heating were initiated on day 4 and delivered for 7 consecutive days. Anti-PD1 (200 μg per dose) was administered intraperitoneally on days 11, 15, 18, 21 and 28. Lung metastases were enumerated on day 20. **A** Experimental schema for TTFields and anti-PD1 combination in the 344SQR subcutaneous model. **B** Tumor growth kinetics. **C** Overall survival. **D** Quantification of lung metastatic nodules. Data are presented as mean ± SEM. NS, not significant; *P < 0.05; **P < 0.01; ***P < 0.001; ****P< 0.0001.

We next asked whether TTFields-mediated tumor conditioning translates into improved ICI sensitivity in vivo using the anti-PD1-resistant murine lung adenocarcinoma cell line 344SQR (Fig. 4A). Consistent with resistance, anti-PD1 monotherapy failed to significantly delay tumor growth compared with control (Fig. 4B). Thermal treatment, administered alone or in combination with anti-PD1, conferred no measurable benefit. TTFields monotherapy transiently delayed tumor progression; however, tumors ultimately escaped control. In contrast, concomitant treatment of TTFields and anti-PD1 induced robust and sustained tumor suppression, with significantly reduced tumor volumes relative to all comparator groups across multiple time points, indicating that TTFields overcome intrinsic resistance to PD1 blockade.

Improved tumor control translated into a marked survival advantage (Fig. 4C). Median survival was 20 days in the control group and 21 days with anti-PD1 alone, with no extension observed in heating-based regimens. TTFields monotherapy did not significantly prolong median survival beyond control. By contrast, TTFields together with anti-PD1 extended median survival to 32 days, significantly outperforming all other treatment arms.

To assess systemic disease control, pulmonary metastases were quantified on day 20 (Fig. 4D). The mean number of lung nodules was 48 ± 6 in control mice and 46 ± 5 in anti-PD1-treated mice, confirming the absence of therapeutic activity with checkpoint blockade alone. TTFields monotherapy significantly reduced metastatic burden (21 ± 2 nodules), and the combination of TTFields with anti-PD1 further decreased lung metastases to 12 ± 2 nodules.

Collectively, these findings demonstrate that TTFields function as a potent sensitizing modality that is capable of restoring functional responsiveness to PD1 blockade. Concomitant treatment of TTFields with anti-PD1 significantly enhanced local tumor control, reduced metastatic dissemination, and prolonged survival in an otherwise refractory tumor model.

### Concomitant therapy remodels immune composition toward an effector-favouring balance

**Fig. 5:**
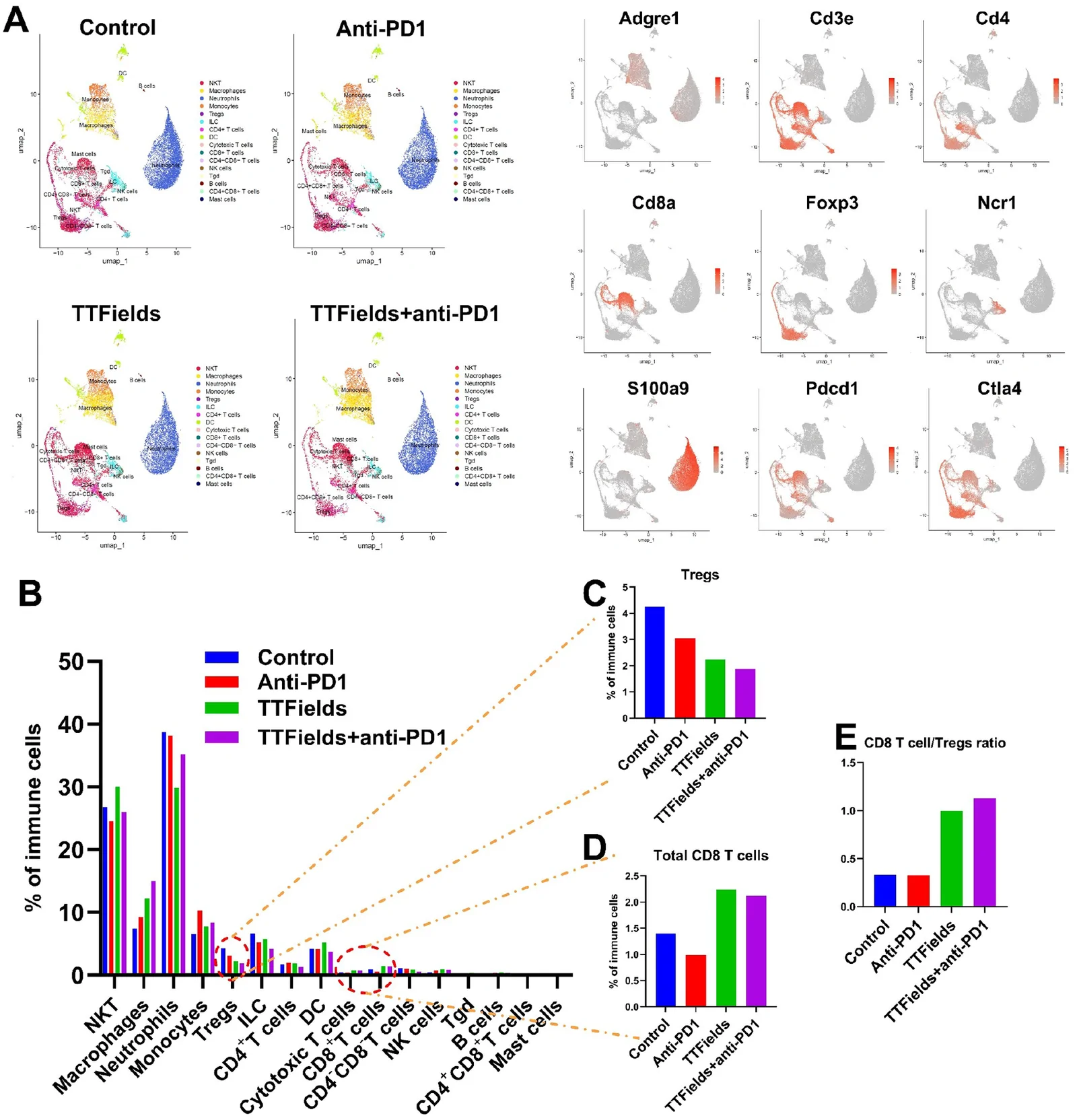
Combination therapy remodels immune composition toward an effector-favouring balance. 344SQR subcutaneous tumors were treated with anti-PD1, TTFields, or the combination and harvested on day 20 after inoculation. Single-cell suspensions were prepared and subjected to 10x Genomics single-cell RNA sequencing to define intratumoral immune composition. **A** UMAP of CD45^+^ immune cells across treatment groups, annotated by canonical lineage-defining markers. **B** Relative frequencies of major immune cell populations within total CD45^+^ cells under each condition. **C** Percentage of Tregs. **D** Percentage of total CD8^+^ T cells. **E** Ratio of CD8^+^ T cells to Tregs.

To delineate the immune mechanisms underlying restored checkpoint sensitivity, tumors from control, anti-PD1, TTFields and TTFields plus anti-PD1 cohorts were harvested on day 20 and subjected to 10x Genomics scRNAseq. Unsupervised clustering resolved major immune compartments across conditions, including CD8^+^ and CD4^+^ T cells, cytotoxic T cells, regulatory T cells (Tregs), NK and NKT cells, B cells, dendritic cells, macrophages, monocytes, neutrophils and innate lymphoid subsets (Fig. 5A, B). Anti-PD1 monotherapy induced minimal changes in global immune composition, consistent with its limited antitumor efficacy. By contrast, TTFields exposure reshaped both lymphoid and myeloid compartments, with the most pronounced remodeling observed in the combination group.

Quantitative analysis revealed that TTFields reduced Tregs from 4.25% in controls to 2.24% following TTFields and to 1.88% with TTFields plus anti-PD1 (Fig. 5C). In parallel, TTFields increased intratumoral CD8^+^ T cell frequency from 1.41% in control tumors to 2.24%, with elevated levels maintained in the combination group (2.12%) (Fig. 5D). Accordingly, the CD8/Treg ratio rose from 0.33 in control and 0.32 in anti-PD1 tumors to 1.0 with TTFields and further to 1.13 with combination therapy (Fig. 5E). Together, these data indicate that, unlike the radiotherapy-associated immune suppression marked by Treg enrichment [19, 20], TTFields reprogram the tumor immune ecosystem away from regulatory dominance and toward effector predominance, thus establishing a cellular framework for restored checkpoint responsiveness and improved tumor control.

### TTFields reprogram macrophage toward enhanced antigen presentation, inflammatory polarization and reduced immunosuppression

**Fig. 6:**
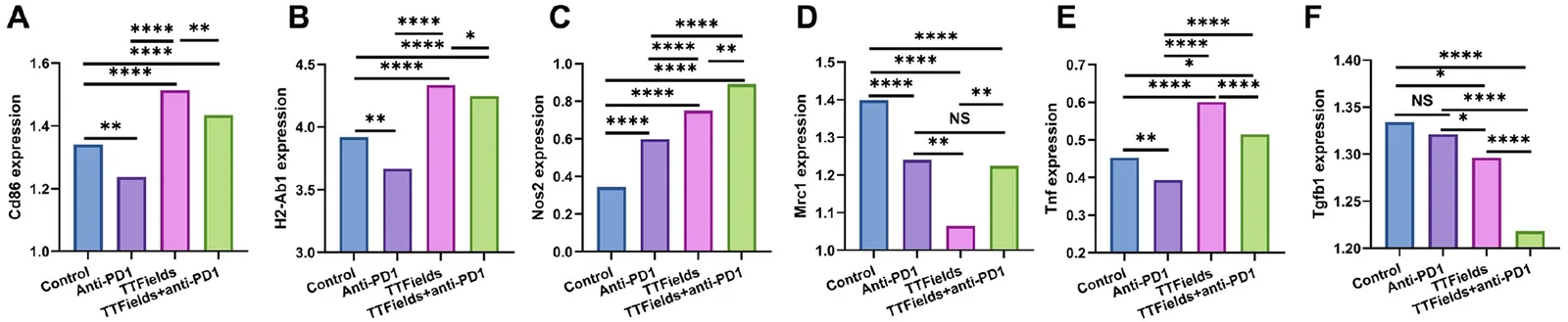
TTFields reprogram macrophages toward a pro-inflammatory and immunostimulatory phenotype in vivo. **A-B** Relative expression of Cd86 and H2-Ab1, indicating enhanced macrophage costimulatory and MHC class II antigen-presenting capacity. **C-D** Relative expression of the M1-like macrophage marker Nos2 and the M2-like macrophage marker Mrc1. **E-F** Relative expression of Tnf and Tgfbl, reflecting inflammatory and immunosuppressive signaling, respectively. NS, not significant; *P < 0.05; **P < 0.01; ***P < 0.001; ****P < 0.0001.

Moving beyond immune composition, we sought to identify a plausible innate mechanism underlying restored checkpoint sensitivity, we next focused on tumor-associated macrophages (TAMs), which represented a prominent myeloid compartment in the 344SQR microenvironment and are well positioned to regulate antigen presentation, cytokine tone and downstream T cell function [16, 18]. Analysis of macrophage-specific transcriptional programs revealed that TTFields induced broad functional reprogramming rather than a simple change in cell abundance (Fig. 6).

First, TTFields enhanced macrophage signatures linked to antigen presentation and co-stimulation. Expression of Cd86 was reduced by anti-PD1 monotherapy relative to control, indicating that PD1 blockade alone did not improve macrophage priming capacity in this resistant setting (Fig. 6A) [21, 22]. By contrast, TTFields significantly increased Cd86 expression relative to both control and anti-PD1, and this increase was largely maintained in the TTFields plus anti-PD1 group, although at a modestly lower level than with TTFields alone. A similar pattern was observed for H2-Ab1, an MHC class II gene associated with macrophage-mediated antigen presentation. Anti-PD1 alone reduced H2-Ab1 relative to control, whereas TTFields significantly increased H2-Ab1 compared with both control and anti-PD1. The combination group likewise retained elevated H2-Ab1 expression relative to control and anti-PD1, again with a modest attenuation compared with TTFields monotherapy. These findings indicate that TTFields, rather than PD1 blockade alone, is the dominant driver of macrophage antigen-presenting and co-stimulatory competence in 344SQR tumors.

We next asked whether this enhancement of antigen presentation was accompanied by polarization toward a more inflammatory macrophage state. TTFields induced a pronounced shift toward M1-like macrophage programming, as evidenced by significant upregulation of Nos2, a canonical inflammatory macrophage marker (Fig. 6B) [23, 24]. Although anti-PD1 monotherapy modestly increased Nos2 relative to control, TTFields further increased Nos2 beyond both control and anti-PD1, and the highest expression was observed in the TTFields plus anti-PD1 group. In parallel, expression of Mrc1 (Cd206), a marker associated with M2-like [23, 24], immunosuppressive macrophages, was significantly reduced by TTFields relative to both control and anti-PD1. Mrc1 remained suppressed in the combination group, without a further decrease beyond that seen with TTFields alone, indicating that TTFields is the primary driver of M2 attenuation. Together, these data show that TTFields shifts macrophages away from an immunoregulatory state and toward an inflammatory, immune-supportive phenotype.

This macrophage repolarization was further reflected in cytokine signaling. TTFields significantly increased expression of the pro-inflammatory cytokine Tnf relative to both control and anti-PD1, consistent with activation of macrophage inflammatory effector programs (Fig. 6C) [25, 26]. The combination group also maintained elevated Tnf expression above control and anti-PD1, although levels were somewhat lower than with TTFields alone. By contrast, the immunoregulatory cytokine Tgfb1 was significantly reduced by TTFields and was most strongly suppressed in the TTFields plus anti-PD1 group [27]. Anti-PD1 alone had little effect on Tgfb1, whereas the combination with TTFields reduced Tgfb1 below control, anti-PD1 and TTFields monotherapy, indicating cooperative relief of macrophage-associated immunosuppressive signaling.

### TTFields plus anti-PD1 drive cytotoxic reprogramming toward a Gzmk-dominant state

**Fig. 7:**
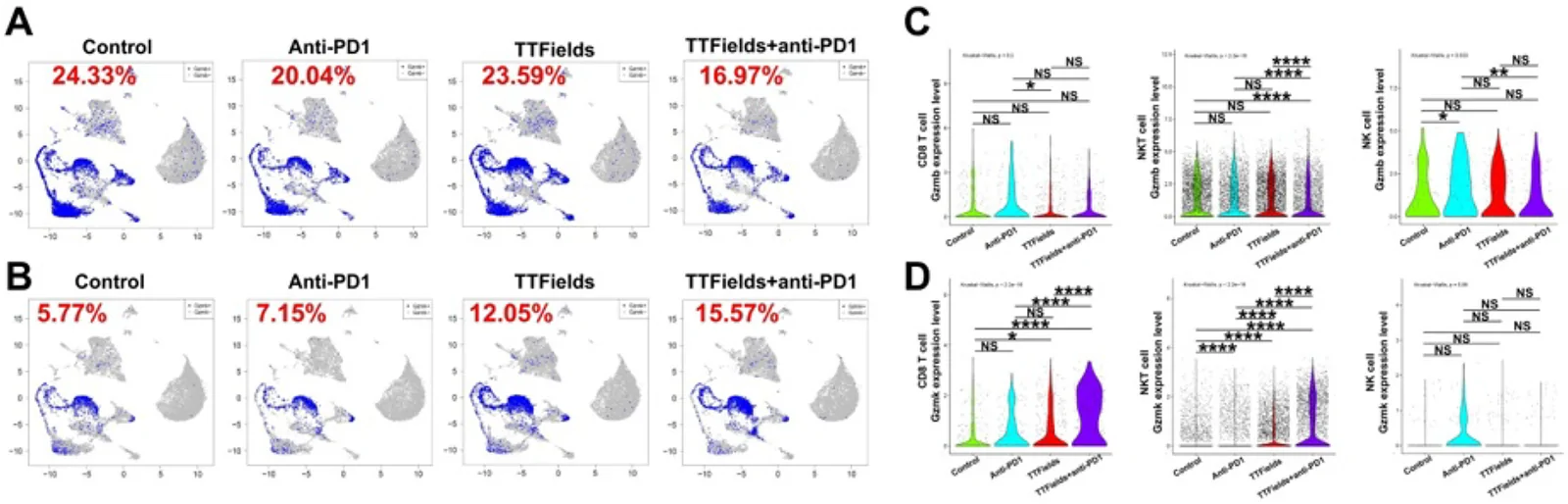
TTFields plus anti-PD1 drive cytotoxic reprogramming toward a Gzmk-dominant state. **A** UMAP projections illustrating the spatial distribution and relative frequency of Gzmb^+^ cells across treatment groups. **B** UMAP projections illustrating the spatial distribution and relative frequency of Gzrnk^+^ cells across treatment groups. **C** Violin plots depicting Gzmb expression in CD8^+^ T cells (left), NKT cells (middle), and NK cells (right) under each treatment condition. **D** Violin plots depicting Gzmk expression in CD8^+^ T cells (left), NKT cells (middle), and NK cells (right) across treatment groups. Gene expression differences were evaluated using the Kruskal-Wallis test with multiple comparisons. A two-sided P < 0.05 was considered statistically significant. *P < 0.05; **P < 0.01; ***P < 0.001; ****p < 0.0001; ns, not significant.

To distinguish numerical expansion from qualitative remodeling of cytotoxic signatures, we profiled granzyme expression across intratumoral lymphocyte subsets. The combination of TTFields and anti-PD1 reduced the overall frequency of Gzmb^+^ immune cells (16.97%) relative to control (24.33%), anti-PD1 (20.04%), or TTFields alone (23.59%) (Fig. 7A). In contrast, Gzmk^+^ cells were markedly enriched in the combination arm (15.57%) compared with control (5.77%), anti-PD1 (7.15%), or TTFields (12.05%) (Fig. 7B), indicating a shift in the dominant cytotoxic signature rather than global amplification of effector programs.

Within CD8^+^ T cells, Gzmb expression did not differ significantly across groups (Fig. 7C), and mean transcript levels remained comparable (control 0.60; anti-PD1 0.88; TTFields 0.51; combination 0.54), arguing against enhanced terminal effector differentiation as the principal mechanism underlying synergy. In contrast, Gzmk expression varied significantly and was maximal in the combination cohort (Fig. 7D). Mean Gzmk increased from 0.38 in control tumors to 0.53 with anti-PD1 and 0.56 with TTFields, reaching 1.21 with combination therapy. Distributional analysis revealed expansion of a high-Gzmk CD8^+^ subset, consistent with selective enrichment of a distinct cytotoxic state rather than uniform transcriptional upregulation.

A similar pattern was observed in NKT cells. Although mean Gzmb expression in the combination group (0.99) was lower than in control (1.27), anti-PD1 (1.25), or TTFields (1.24) tumors (Fig. 7C), Gzmk was substantially elevated, increasing from 0.16 in control to 0.30 with anti-PD1 and 0.35 with TTFields, and reaching 0.80 with combination treatment (Fig. 7D). This reprogramming was selective for TCR-bearing lymphocytes. NK cells exhibited reduced Gzmb expression in the combination arm relative to anti-PD1 alone (1.05 versus 1.61) without a corresponding increase in Gzmk, and ILC subsets showed no significant transcriptional changes (Supplementary Fig. 2).

Collectively, these data indicate that TTFields combined with PD1 blockade does not potentiate canonical Gzmb-driven terminal cytotoxicity [28]. Instead, it promotes a Gzmk-dominant effector program within CD8^+^ T and NKT cells, consistent with qualitative cytotoxic reprogramming as a mechanistic basis for restored checkpoint responsiveness[29, 30].

### TTFields combined with anti-PD1 attenuate inhibitory checkpoint architecture in lymphocytes

To assess whether TTFields-based therapy remodels inhibitory receptor landscapes, we quantified Pdcd1 and Ctla4 expression across CD8^+^ T cells, CD4^+^ T cells and NKT cells by scRNAseq.

**Fig. 8:**
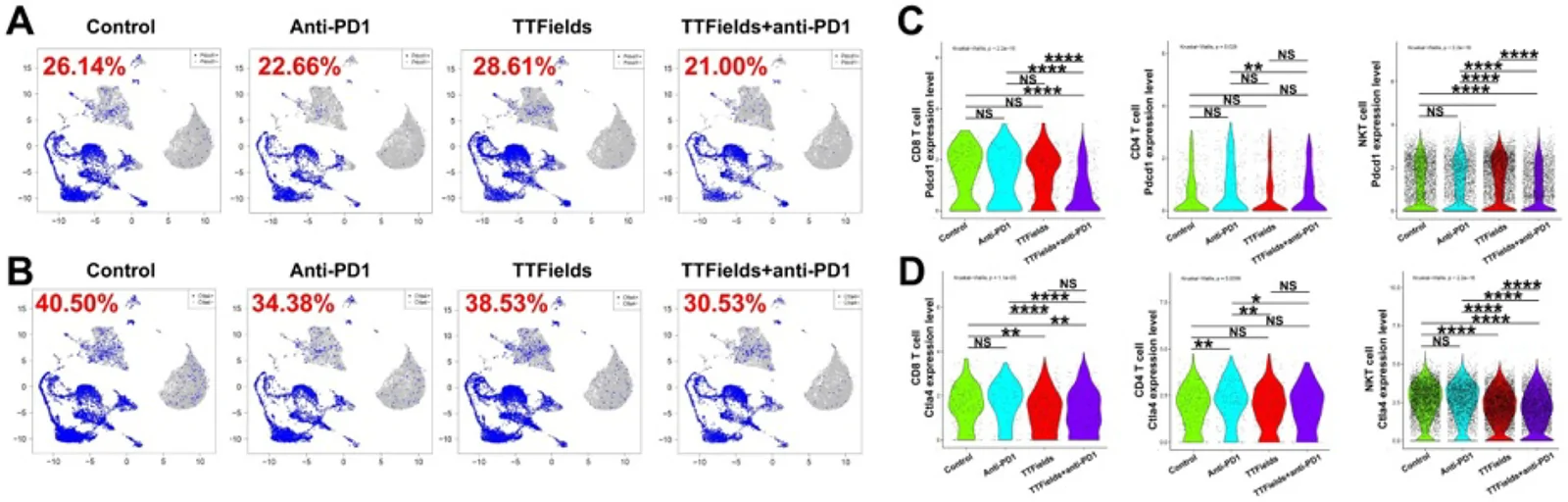
TTFields combined with anti-PD1 attenuate inhibitory checkpoint architecture in lymphocytes. **A** UMAP projections showing the spatial distribution and relative abundance of Pdcd1^+^ cells across treatment groups. **B** UMAP projections showing the spatial distribution and relative abundance of Ctla4^+^ cells across treatment groups. C Violin plots of Pdcd1 expression in CD8^+^ T cells (left), CD4^+^ T cells (middle), and NKT cells (right) under each treatment condition. **D** Violin plots of Ctla4 expression in CD8^+^ T cells (left), CD4^+^ T cells (middle), and NKT cells (right) across treatment groups. Gene expression differences were assessed using the Kruskal-Wallis test with correction for multiple comparisons. All tests were two-sided; P < 0.05 was considered statistically significant. *P < 0.05; **P < 0.01; ****p < 0.0001; NS, not significant.

At the global immune-cell level, TTFields combined with anti-PD1 reduced the frequency of Pdcd1^+^ cells (21.00%) compared with control (26.14%), anti-PD1 alone (22.66%) and TTFields monotherapy (28.61%) (Fig. 8A). Similarly, the combination arm exhibited a lower proportion of Ctla4^+^ cells (30.53%) relative to control (40.53%), anti-PD1 (34.38%) and TTFields alone (38.53%) (Fig. 8B), indicating contraction of inhibitory checkpoint-expressing populations.

In CD8^+^ T cells, Pdcd1 expression differed significantly among treatment groups (Fig. 8C). Anti-PD1 monotherapy (1.31) modestly reduced Pdcd1 relative to control (1.43), whereas TTFields alone maintained comparable levels (1.41). Notably, the combination of TTFields with anti-PD1 (0.76) resulted in a significant reduction in Pdcd1 compared with control, anti-PD1 alone and TTFields monotherapy, indicating attenuation of PD1-associated inhibitory signaling under dual treatment. A similar pattern was observed for Ctla4 (Fig. 8D). Although expression varied across conditions, combination therapy (1.32) significantly reduced Ctla4 relative to control (1.56) and anti-PD1 monotherapy (1.69). These data indicate that TTFields plus anti-PD1 suppress key inhibitory checkpoint transcripts in CD8^+^ T cells rather than reinforcing exhaustion-associated states.

In CD4^+^ T cells, modulation was more modest but directionally consistent. Combination therapy reduced Pdcd1 expression (0.59) relative to anti-PD1 monotherapy (0.76) (Fig. 8C) and similarly decreased Ctla4 (1.83 versus 2.13) (Fig. 8D), without evidence of coordinated upregulation suggestive of terminal exhaustion.

In contrast, NKT cells demonstrated more pronounced checkpoint remodeling. Both Pdcd1 and Ctla4 differed significantly across groups. Combination therapy reduced Pdcd1 expression (0.94) relative to control (1.10), anti-PD1 (1.13) and TTFields alone (1.28) (Fig. 8C). Likewise, Ctla4 expression was lower in the combination arm (1.92) compared with control (2.49), anti-PD1 (2.49) and TTFields monotherapy (2.07) (Fig. 8D), indicating a broader attenuation of inhibitory receptor expression in this compartment.

Collectively, these findings demonstrate that TTFields combined with PD1 blockade does not promote terminal exhaustion. Instead, it selectively dampens PD1 and CTLA4 expression within CD8^+^ T cells, CD4^+^ T cells and NKT cells, consistent with a checkpoint-rebalanced and functionally permissive cytotoxic state. Integrated with increased CD8^+^ T cell representation and expansion of Gzmk^+^ effector subsets, these data support a model in which TTFields restores antitumor immunity through coordinated attenuation of inhibitory receptor architecture rather than escalation of exhaustion-associated pathways.

### TTFields cooperates with PD1 blockade to remodel the intratumoral TCR repertoire and promote dominant clonal expansion

**Fig. 9:**
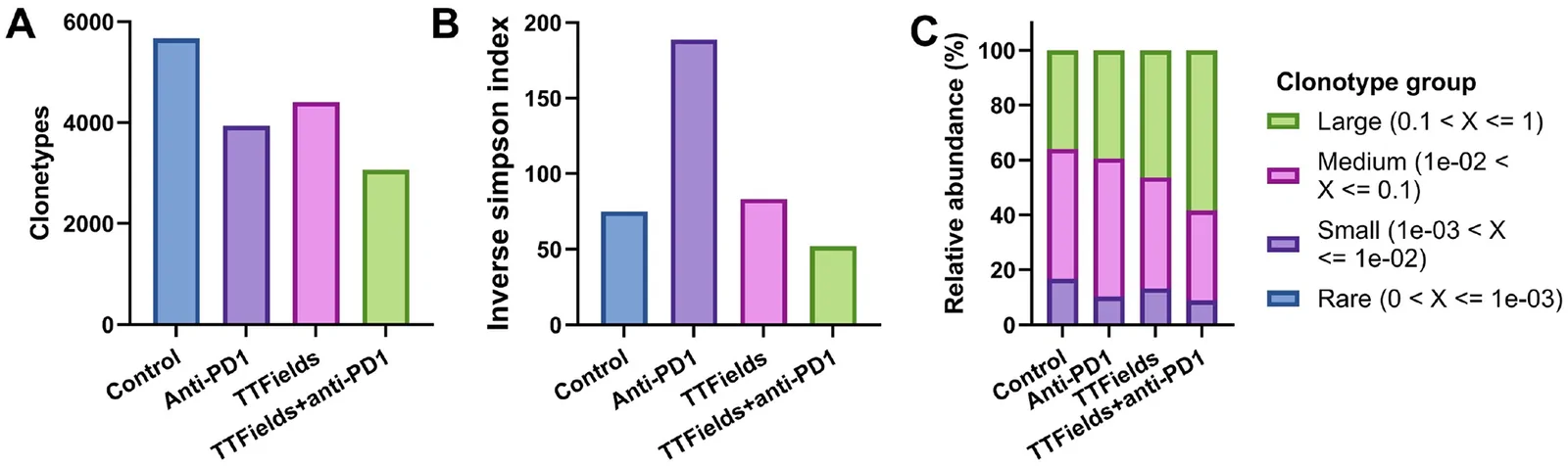
TTFields and PD-1 blockade reshape the intratumoral TCR repertoire and promote dominant clonal expansion. TCR repertoires were analyzed across treatment groups to evaluate clonotype richness, clonal composition and diversity. **A** Total number of unique TCR clonotypes detected in each treatment group. **B** TCR repertoire diversity estimated using the inverse Simpson index. C Distribution of clonotype frequencies stratified into rare (0 < × ≤ 10^−5^), small (10^−5^ < × ≤ 10^−4^), medium (10^−4^ < × ≤ 0.001) and large (0.001 < × ≤ 1) abundance categories.

To investigate how TTFields modulate adaptive immune responses to checkpoint blockade, we profiled the tumor-infiltrating T cell receptor (TCR) repertoire across treatment groups and assessed clonotype richness, clonal distribution and repertoire diversity. Quantification of unique TCR clonotypes revealed substantial differences in repertoire breadth among treatments (Fig. 9A). Control tumors exhibited the greatest clonotype richness, with approximately 5668 unique clonotypes, consistent with a highly diverse but weakly expanded T cell population. PD1 blockade reduced the total number of unique clonotypes to 3938, whereas TTFields monotherapy yielded an intermediate repertoire size (4405 clonotypes). Notably, tumors treated with TTFields combined with PD1 blockade showed the lowest clonotype richness (3069), indicating contraction of repertoire breadth accompanied by preferential expansion of selected T cell clones.

This refined redistribution was further reflected in diversity metrics. PD1 blockade alone generated the highest repertoire diversity, with an inverse Simpson index of 189, consistent with expansion of a relatively broad set of clonotypes (Fig. 9B). TTFields monotherapy produced a more modest increase relative to control (83 versus 75). In contrast, the combination of TTFields and PD1 blockade yielded the lowest diversity, with an inverse Simpson index of 52, indicating a repertoire dominated by a restricted number of highly expanded clones, potentially refined to be more tumor-targeting.

We next examined the distribution of clonotype frequencies by stratifying clones into rare, small, medium and large abundance categories (Fig. 9C). Control tumors displayed a relatively balanced distribution dominated by medium-frequency clonotypes, with large clones comprising 35.95% of the repertoire. PD1 blockade modestly increased the proportion of expanded clones (39.48% large clonotypes). In contrast, TTFields + anti-PD1 treatment markedly shifted the repertoire toward dominant clonal expansion, with large clonotypes accounting for 58.36% of all TCRs, accompanied by a reduction in medium-frequency clones. TTFields monotherapy also increased the fraction of large clones (46.37%) compared with control, although this effect was less pronounced than with the combination therapy.

Together, these findings demonstrate that PD1 blockade predominantly broadens the intratumoral T cell repertoire, whereas TTFields synergizes with checkpoint inhibition to drive focused expansion of dominant T cell clonotypes. The resulting shift toward oligoclonal T cell dominance is consistent with antigen-driven expansion of tumor-reactive T cells and suggests that TTFields enhances the capacity of PD1 blockade to promote effective antitumor T cell responses.

## Discussion

Immune resistance in cancer is not maintained by a single lesion, but by coordinated defects in tumor-cell fitness, innate priming and adaptive lymphocyte function. Here we show that TTFields restore sensitivity to PD1 blockade in an anti-PD1-refractory lung adenocarcinoma model by remodeling each of these compartments. TTFields alone directly suppressed tumor-cell proliferation and clonogenic survival, but their dominant therapeutic effect in vivo emerged when combined with PD1 blockade, where they produced sustained tumor control, reduced pulmonary metastases and prolonged survival. These findings identify TTFields as a tumor-conditioning modality capable of converting a checkpoint-refractory immune microenvironment into one that supports productive antitumor immunity.

Our data extend the relevance of previous studies showing that TTFields have biological effects beyond mitotic disruption. TTFields are known to interfere with spindle assembly, cytokinesis and organelle localization in dividing tumor cells [11]. More recent work has shown that TTFields can induce immunogenic cell death, activate STING and AIM2 inflammasome signalling, and enhance antitumor immunity when treated concomitantly with PD1 blockade [12, 14]. Consistent with these reports, we found that TTFields induced dynamic tumor-cell transcriptional reprogramming and immune remodelling that restored responsiveness to anti-PD1. Thus, TTFields should not be viewed solely as a cytostatic or antimitotic intervention, but also as a biophysical immune-conditioning strategy.

A notable feature of the tumor-cell response was its temporal plasticity. TTFields produced maximal tumor growth inhibition and clonogenic suppression at 48 h, followed by partial recovery at 72 h. This rebound was accompanied by reactivation of cell-cycle, metabolic and interferon-associated transcriptional programs. These findings suggest that prolonged TTFields exposure alone imposes selective or adaptive pressure rather than producing monotonically increasing tumor-cell suppression. Such adaptation may explain why TTFields monotherapy delayed tumor progression but did not achieve durable control. In contrast, when TTFields-induced tumor stress was paired with PD1 blockade, this transient conditioning state was converted into sustained therapeutic benefit.

The in vivo findings support this interpretation. Anti-PD1 alone had minimal activity in the 344SQR model, consistent with its established checkpoint-refractory phenotype [15]. Heating controls were inactive, indicating that the therapeutic effect was not attributable to nonspecific thermal stress. By contrast, TTFields plus anti-PD1 produced durable tumor suppression, reduced metastatic burden and improved survival. Prior work has shown therapeutic activity for TTFields combined with checkpoint inhibition in preclinical NSCLC models [13]. Our study advances this concept by demonstrating efficacy in an anti-PD1-resistant model and by defining the associated immune mechanisms at single-cell resolution.

Single-cell profiling showed that TTFields shifted the tumor immune microenvironment away from regulatory dominance. TTFields reduced Treg abundance, increased CD8^+^ T cell representation and markedly increased the CD8^+^ T cell-to-Treg ratio. This is mechanistically relevant because suppressive regulatory populations can constrain checkpoint efficacy even when effector T cells are present [2–4]. The direction of this effect also contrasts with some radiation-induced immune responses, in which local tumor perturbation can be accompanied by compensatory Treg enrichment and TGF-β-associated suppression [10, 19, 20]. Thus, whereas radiotherapy can generate both immune-stimulatory and immune-suppressive signals [6, 7, 9], TTFields in this model relieved regulatory pressure while preserving or enhancing effector lymphocyte representation.

Macrophage reprogramming we observed provides a plausible upstream mechanism for this immune shift. TTFields increased Cd86 and H2-Ab1 expression, indicating enhanced co-stimulatory and antigen-presenting capacity. TTFields also increased Nos2 and Tnf while reducing Mrc1 and Tgfb1, consistent with polarization away from an immunoregulatory macrophage state and toward an inflammatory, immune-supportive phenotype [21–23, 25–27]. Because tumor-associated macrophages regulate antigen presentation, cytokine tone, T cell recruitment and immune suppression, this innate remodelling is likely central to the restoration of checkpoint sensitivity. The further reduction of Tgfb1 in the combination group suggests cooperation between TTFields and PD1 blockade in relieving macrophage-associated immunosuppression.

The suppression of Tgfb1 is particularly important. TGF-β signalling restricts antitumor immunity through multiple mechanisms, including impaired antigen presentation, T cell exclusion, Treg differentiation, stromal remodelling and myeloid suppression [10, 27]. In the radiotherapy setting, TGF-β has been identified as a major barrier to radiation-induced antitumor immunity [10]. In our model, TTFields reduced macrophage Tgfb1 expression, and this effect was strongest with TTFields plus anti-PD1. These findings position attenuation of macrophage-associated TGF-β signalling as a potential mechanism by which TTFields create a more permissive environment for PD1 blockade.

At the lymphocyte level, TTFields plus anti-PD1 promoted qualitative cytotoxic reprogramming rather than simple amplification of terminal effector activity. Combination therapy did not enhance a canonical Gzmb-dominant program. Instead, it enriched a Gzmk-dominant state in CD8^+^ T cells and NKT cells. This distinction is important because intratumoral CD8^+^ T cells occupy a continuum of functional states, including progenitor-like, transitory, cytotoxic and terminally exhausted populations [28, 29]. Checkpoint blockade preferentially acts on specific exhaustion-associated subsets rather than uniformly reinvigorating all dysfunctional T cells [28]. The emergence of Gzmk-dominant CD8^+^ T and NKT cells therefore suggests that TTFields plus anti-PD1 favours a cytotoxic but non-terminal lymphocyte state compatible with checkpoint responsiveness [29, 30].

This interpretation is reinforced by the inhibitory receptor analysis. TTFields plus anti-PD1 reduced Pdcd1 and Ctla4 expression across CD8^+^ T cells, CD4^+^ T cells and NKT cells, without evidence of coordinated terminal exhaustion. Thus, TTFields did not simply increase lymphocyte activation at the cost of deeper exhaustion. Instead, combination therapy rebalanced lymphocyte state by increasing effector-favouring cytotoxic features while attenuating inhibitory checkpoint architecture. Together with the increased CD8^+^ T cell-to-Treg ratio and enhanced macrophage antigen-presentation program, these changes provide a cellular basis for restored PD1 sensitivity.

TCR profiling further supports an antigen-driven adaptive immune mechanism. Anti-PD1 monotherapy broadened repertoire diversity but did not control tumor growth, indicating that repertoire breadth alone was insufficient in this resistant setting. In contrast, TTFields plus anti-PD1 reduced clonotype diversity while increasing the dominance of selected clones. This oligoclonal pattern is consistent with focused expansion of selected tumor-reactive T cell populations. Similar TTFields-associated T cell activation and clonal expansion signatures have been reported in glioblastoma samples [12]. Our data suggest that, in resistant tumors, TTFields convert PD1 blockade from a broad but ineffective stimulus into a focused clonal expansion program associated with tumor control.

## Conclusions

In summary, these findings support a model in which TTFields restore checkpoint sensitivity through coordinated tumor-immune remodelling. TTFields first impose tumor-cell stress and suppress proliferative fitness. This is accompanied by macrophage reprogramming toward antigen presentation, co-stimulation, inflammatory polarization and reduced Tgfb1-associated suppression. The immune microenvironment then shifts away from Treg dominance toward a higher CD8^+^ T cell-to-Treg ratio. In this context, PD1 blockade promotes Gzmk-dominant cytotoxic lymphocyte states, attenuates inhibitory receptor expression and drives focused T cell clonal expansion. This integrated mechanism distinguishes TTFields from approaches that rely primarily on direct tumor cytotoxicity or single-axis immune modulation.

## Supplementary Material

This is a list of supplementary files associated with this preprint. Click to download.

- Supplementarymaterials.docx

## Data Availability

The data and materials supporting the findings of this study are available from the corresponding author upon reasonable request.

## Abbreviations

CTLA4: Cytotoxic T-lymphocyte-associated protein 4
GSEA: Gene set enrichment analysis
ICI: Immune checkpoint inhibitor
ILC: Innate lymphoid cell
MHC: Major histocompatibility complex
NES: Normalized enrichment score
NSCLC: Non-small-cell lung cancer
PD1: Programmed cell death protein 1 RNA-seq RNA sequencing
scRNA-seq: Single-cell RNA sequencing
STING: Stimulator of interferon genes
TAM: Tumor-associated macrophage
TCR: T-cell receptor
Treg: Regulatory T cell
TTFields: Tumor Treating Fields
